# Effectiveness of timed and non-timed second appointments in improving uptake in breast cancer screening

**DOI:** 10.1177/0969141315624937

**Published:** 2016-03-02

**Authors:** Sue Hudson, Debbie Brazil, William Teh, Stephen W Duffy, Jonathan P Myles

**Affiliations:** 1 Peel and Schriek Consulting Limited, UK; 2 Department of Surgical Services, Luton and Dunstable Hospital, Luton, UK; 3 North London Breast Screening Service, London, UK; 4 Centre for Cancer Prevention, Wolfson Institute of Preventive Medicine, Barts and the London School of Medicine and Dentistry, Queen Mary University of London, London, UK

**Keywords:** Breast cancer screening, non-attendance, second timed appointments

## Abstract

**Objectives:**

To estimate the effect on breast screening uptake of delayed, targeted, second timed appointments in women who did not take up an initial breast cancer screening appointment offer.

**Methods:**

Non-attending women received a four-month delayed second timed appointment following non-response to the initial invitation and the normal open invitation sent to non-attenders. A comparison group was sent a four-month delayed additional open invitation.

**Results:**

Response to the second timed appointments (percentage of re-invited women eventually attending in this episode) was 20%, corresponding to an estimated increase on total uptake of 6%. Response was highest in women who had previously attended screens. Response in the women offered an additional delayed open invitation was 7.5%, corresponding to an estimated 2.3% increase in overall uptake.

**Conclusions:**

Second timed appointments were almost three times as effective as additional open invitation. They should be targeted at women most likely to attend. A randomized study of second timed appointments versus open invitations should be conducted.

## Introduction

Screening for breast cancer has been shown to reduce deaths due to the disease, although the magnitude of side-effects such as over-diagnosis remains uncertain.^1^ In Britain, as part of the NHS Breast Screening Programme, women aged 50–70 are invited by post to attend for two-view mammography every three years, with extension to age groups 47–49 and 71–73 currently being piloted nationally. National uptake in 2007/2008 and 2010/2011 was 73.2% and 73.4%, but in London these figures were 60.6% and 63.6%, respectively. The lower uptake in London is thought to be due in part to the increased population mobility and, therefore, women not receiving their invitations,^2^ lower socioeconomic status and lower uptake in certain ethnic groups.^3–7^

Previous work^8^ has suggested that participation can be increased by sending women who do not attend their first appointment (‘DNA women’) a second timed appointment (2TA) (i.e., an invitation to a screening appointment at a specific date and time, as in the initial invitation) as opposed to an ‘open invitation’ (an invitation to contact the screening centre and arrange an appointment). The authors therefore evaluated the use of 2TAs by conducting a study in which women were sent such an invitation, and the response rate observed. In a second study, DNA women were sent a standard open invitation. As a large number of second invitations will be missed, wasting both time and resources, it was also of interest to examine the effectiveness of targeting invitations to particular groups of women (in terms of their age, whether they were being invited to prevalent (first) or incident (subsequent) screens, and in the case of the latter, the length of time since the woman last attended a screen).

The authors also investigated the effectiveness of sending second invitations at an interval of four months after the original invitation. The hypothesis was that this may improve ultimate uptake in areas of high mobility (because the centre administering the system may, by then, have the correct address of a woman who has recently moved).

## Methods

The normal screening invitation process offers women a timed and dated appointment within 36 months of their previous screen (or before their 53rd birthday for first invitation). Women who do not attend the appointment (and do not contact the office to cancel or rebook) are sent an open invitation to call and make an invitation at any time. This project, consisting of two studies, estimates the impact of re-inviting the cohort that does not respond to this process at a further four months after their screening episode was initially opened.

In the first study, 2439 women invited for screening by the North London Breast Screening Service (NLBSS) were entered. The cohort was made up of all women from screening batches opened during the period June to August 2010, who subsequently did not attend their screening appointment at one of three screening locations in North London. These women were offered a 2TA between late October and late November 2010, to induce a delay between their first and second invitations while still providing for them to be screened within six months of the episode opening date, the usual definition of attendance in the programme. The response rate was calculated as the number of 2TA women who attended within six months of the opening of their episode (the date the list of invitees was compiled) as a proportion of all women in the 2TA cohort. The corresponding increase in overall uptake was estimated, based on the total number of women invited across the whole NLBSS in the same period. The response rate of the 2TA cohort women was analysed by stratified groups, to identify populations which might benefit most from the intervention. In a second study, all non-attenders from November 2010 were sent a four-month delayed open invitation, and their response rate calculated six months later and analysed in a similar way to the first study.

Ethical approval for these studies was not sought because they constituted evaluation of service modifications with no additional clinical intervention or sharing of individual information.

## Results

Of the 2439 women offered a 2TA, 488 attended an appointment before the end of the episode, giving a response rate of 20%. They did not always attend the 2TA on the date and time offered; some rebooked a different appointment, which may have been after the six-month period. The overall response rate to the additional open invitation sent to non-attenders was 8% (160 women attended within six months of their open episode date, out of 2127 open invitations sent), which was significantly less than 2TAs (*p* < 0.01), although women were not randomized between 2TAs and open appointments.

For the women receiving 2TAs, additional information on the age and screening history is available. Table 1 shows the number of women invited, and response to a 2TA, divided into three groups:

1. 501 women aged under 53 as a proxy for first invitation (of these, 38 women had had a previous screen);
2. 938 women aged 53–70 who had previously attended screening (known as incident screening episodes);
3. 1000 women aged 53–70 who had been invited before, but never previously attended screening (known as ‘prevalent’ screening episodes).

**Table 1.** Uptake of 2TAs by screening episode subgroup.

Groups of women who received 2TA	Number of women receiving 2TA	Number attending 2TA	Response rate	Cancellation rate	Proportion of total screening population in this group	Estimated impact on overall service uptake
Group I (under 53)	501	120	24%	15%	6%	1.5%
Group 2 (53+ and incident)	938	279	30%	20%	12%	3.4%
Group 3 (53+ and prevalent)	1000	89	9%	10%	12%	1.1%
Total in women receiving 2TAs	2439	488	20%	15%	30%	6.0%
Women receiving open appointments	2127	160	8%	NA	30%	2.4%

2TA: second timed appointment.

Table 1 also shows the response rate to open invitations.

Group 2 has the highest response rate amongst women receiving 2TAs, and Group 3 the lowest. Women in Group 3, as well as having the poorest response (attendance) rate, were also the least likely to cancel the appointment (allowing them to be reused), and hence wasted proportionately more appointments (by definition, open appointments cannot be cancelled).

These figures can be used to obtain estimates of the impact on overall uptake, i.e. the absolute increase in attendance rate, of sending 2TAs to a particular group. For example, non-responders under age 53 comprise 6% of the population in the 2TA study and the response to the 2TAs in this subgroup is 24% = 0.24, so we can conclude that sending 2TAs to women in this group would result in an absolute increase in uptake of (0.24 × 6%) = 1.5%. Overall, non-responders were 30% of the eligible population, indicating an overall increase of 6% (20% × 30% = 6%). Assuming the same proportion of initial non-responders for the open invitation, the overall effect would be 8% of 30%, i.e., 2.4%. These figures can be used to calculate the percentage of extra invitations sent, the uptake, and the increase in uptake under three scenarios: inviting Group 1 women only, inviting Groups 1 and 2 women only and inviting all women.

This indicates that the most efficient approach to increasing service uptake can be achieved by limiting 2TAs to Groups 1 and 2, which deliver an estimated 4.9% (=1.5% + 3.4%, from the first two rows of Table 1) improvement, and necessitate an extra 18% = (= 6% +12%) additional invitations. Group 3 only delivered 1% improvement, but would require a further 12% invitations.

Table 2 shows the response rate for 2TAs and open appointments for incident women, broken down by length of time since their previous attended screens. Women who last attended between one and three years ago had the highest response rate to a 2TA at 43%, which is almost twice the rate of those screened between six and nine years ago. Those screened between six and nine years ago have a similar response rate to that of women aged under 53 in the prevalent round. A similar pattern, though with smaller response rates, is noted in the response to second open appointments.

**Table 2.** Attendance rate incident women by time since previous attended screen.

	Second timed appointments			Open appointments		
Time since previous attended screen	Number in group	Number attending	Response rate	Number in group	Number attending	Response rate
Last screen <1 year	24	3	13%	30	5	17%
1 to <3 years	336	146	43%	441	66	15%
3 to <6 years	181	69	38%	96	14	15%
6 to <9 years	213	49	23%	150	9	6%
9+ years	129	15	12%	113	4	4%
No screening history available	93	14	15%			
Total	976	221	30%	830	98	12%

## Discussion

We found that four-month delayed 2TAs sent to non-attenders had nearly three times the response rate in comparison with additional four-month delayed open invitations (20% vs. 7.5%), which yielded a correspondingly larger increase in service-wide uptake. A limitation of the study is that this comparison does not pertain to a randomized trial, but the results are plausible, and compatible, in qualitative terms, with a previous trial.^8^ The results also show a diminishing return of second invitation with time since last attendance for screening. This is also consistent with the results of Stead et al.,^8^ but absolute numbers and magnitudes of effect differ (e.g., the Stead et al. study did not include a delay before sending 2TAs and found a response of 22.8% to 2TAs compared with 12.3% in open appointments). These results suggest that a definitive new randomized study is indicated, as the social and occupational status of the target population for screening has changed since 1998.^9^

As noted above, previous attenders and first invitees are most likely to attend, as has been observed in the programme, and are most likely to respond to the 2TA initiative. In addition, decreasing returns from 2TAs were seen with increasing time since last attending for screening.

Based on the findings from this study, 2TAs were implemented at the NLBSS, and routine processes were changed. For reasons of cost-effectiveness, 2TAs were initially introduced for all invited women who had attended a screen in the last six years (a subset of Group 2), and all women aged under 53 (Group 1), because it was a proxy for first invite (they generally had no screening history to assess attendance behaviour). Selecting these women and administrating their letters was a time-consuming manual process, which involved added stationery and postage costs. In addition, there was a need to minimize waste on clinic capacity.

In terms of inequalities, non-attenders are likely to be of lower socioeconomic status than attenders, so there is at least a potential to reduce social inequalities in delivery. However, previously screened women, who are more likely to benefit from 2TAs, are also more likely to be of higher socioeconomic status than never-screened women, so the use of 2TAs may not, on its own, address inequalities in participation. Despite this, the development and automation of a system for allocating second timed or open appointments, with prior screening attendance history as one of the criteria, is potentially productive in both public health and economic terms. A cost-effectiveness analysis will follow, with the results of the randomized trial, which is in progress.

During the course of this study, and the subsequent preparation of the manuscript, NHS screening policy in England has changed, so that all non-attendees are recommended to be sent a 2TA (although it is not clear that this is universally practised). This study indicates that, for many non-attenders, this will be of benefit; however, by sending 2TAs to all women, rather than focusing on women most likely to attend, our research suggests that valuable resources are being under-utilized. A possible compromise might be to send 2TAs to all women, but to ‘overbook’ those women least likely to attend. Our research also suggests the effectiveness of sending the second appointment, whether timed or not, a few months after the initial invitation, rather than immediately after, to make contact with women who have recently moved, although we did not have a control group who were invited immediately after non-attendance.
